# Tunable blue-green-emitting wurtzite ZnS:Mg nanosheet-assembled hierarchical spheres for near-UV white LEDs

**DOI:** 10.1186/1556-276X-9-20

**Published:** 2014-01-13

**Authors:** Devulapalli Amaranatha Reddy, Deok Hyeon Kim, Seuk Joo Rhee, Bo Wha Lee, Chunli Liu

**Affiliations:** 1 Department of Physics, Hankuk University of Foreign Studies, Gyeonggi 449-791, Republic of Korea

**Keywords:** ZnS:Mg, Hierarchical spheres, Wurtzite structure, Bandgap expansion, Tunable blue-green emission

## Abstract

Mg-doped ZnS hierarchical spheres have been synthesized via hydrothermal method using mixed solvents of ethylenediamine and DI water without any surface-active agent. The surface morphology and microstructure studies revealed that the hierarchical spheres were consisted of many well-aligned nanosheets with width 10 nm and length about 50 ~ 100 nm. X-Ray diffraction results show that the ZnS:Mg hierarchical spheres have wurtzite structure with high crystallinity. The absorption edge in the diffuse reflection spectra shifts towards lower wavelength with increasing Mg concentration, indicating an expansion in the bandgap energy that is estimated to be in the range of 3.28 to 3.47 eV. Blue-green photoluminescence with tunable intensity and peak position was observed depending on the Mg content. The Mg^2+^-activated ZnS phosphor can be good candidates for blue-green components in near-UV white light-emitting diodes.

## Background

In recent years, there is an explosive development of inorganic semiconductor nanostructures, particularly low-dimensional nanostructures. A variety of low-dimensional nanostructures such as zero-dimensional (0D) nanoparticles; one-dimensional (1D) nanowires, nanotubes, nanorods, and nanobelts; and two-dimensional (2D) nanosheets are investigated extensively due to their novel and fascinating properties compared to their bulk counterparts [1-3]. In addition, as the dimension of a material is reduced to the nanometer scale level, a large percentage of atoms are located at the surface, which significantly affects the structural and optical properties. The surface defects decorating the nanostructures of compound semiconductors often give rise to a rich visible luminescence that is attractive for applications in optical devices [1-4]. However, when the individual semiconductor devices are connected together to form integrated optical or electronic devices, the non-chemical connections between the units limit their cooperative or collective physical responses because of the multi-boundaries of electronic states [5]. Hence, complicated nanostructures such as hierarchical, tetrapod, branched, and dendritic structures with natural junctions between branches or arms are highly desired for interconnection applications in the bottom-up self-assembly approach towards future nanocircuits and nanodevices [5].

Among all inorganic semiconductors, ZnS is one important electronic and optoelectronic material with prominent applications in visible-blind UV-light sensors [6,7], gas sensors [8], field-emitters [9], piezoelectric energy generation [10], bioimaging [11], photocatalyst in environmental contaminant elimination [12], H_2_ evolution [13], CO_2_ reduction [14], determination of nucleic acids [15], solar cells [16], infrared windows [17], optical devices [18], light-emitting diodes [19], lasers [20], logic gates, transistors, etc. [2]. ZnS has a bandgap energy of 3.72 eV for its cubic sphalerite phase and 3.77 eV for the hexagonal wurtzite phase [2]. It is well known that at room temperature, only the cubic ZnS is stable, and it can transform to the hexagonal phases at about 1,020°C [2]. For optoelectronics, wurtzite ZnS is more desirable because its luminescent properties are considerably enhanced than sphalerite [21]. Attempts have been reported for preparation of wurtzite ZnS and related materials at lower temperatures through nanoparticle size control or surface-modifying reagents. However, achieving pure-phased wurtzite ZnS with structural stability at ambient conditions remains a challenging issue [22].

Luminescent properties can be significantly enhanced when suitable activators are added to phosphors. The choice of dopant materials and method of preparation have a crucial effect on the luminescence characteristics. Up to now, various processing routes have been developed for the synthesis and commercial production of ZnS nanophosphors, such as RF thermal plasma [23], co-precipitation method [24], sol-gel method [25], and hydrothermal/solvothermal method [26]. The hydrothermal technique is simple and inexpensive, and it produces samples with high purity, good uniformity in size, and good stoichiometry. To prepare ZnS-based high-efficiency luminescent phosphors, transition metal and rare earth metal ions have been widely used as dopants [27-32]. However, studies on the effect of alkaline metal ions doping on the properties of ZnS are sparingly available except few reports on cubic structured ZnS nanostructures [33-35]. In this work, we report on the lower temperature synthesis of stable Mg-doped ZnS wurtzite nanostructures using hydrothermal technique and their luminescence properties. Mg was chosen as the dopant material because it has comparable ionic radius with Zn and has been used as an environment-friendly phosphor for many applications [36-39]. No report is available on wurtzite Mg-doped ZnS nanostructures despite of the importance of ZnS. In the present work, a systematic investigation was carried out on the effect of Mg doping on the structural, optical, and photoluminescence properties of ZnS:Mg nanostructures.

## Methods

Zn_1−*x*_Mg_*x*_S (*x* = 0.00, 0.02, 0.03, 0.04, and 0.05) were prepared using hydrothermal method. In a typical synthesis, Zn(CH_3_COO)_2_ · 2H_2_O, CH_4_N_2_S, and Mg(CH_3_COO)_2_ were dissolved according to stoichiometry into a solution of ethylenediamine (EN) 30 ml and DI water (70 ml). The reaction was carried out at room temperature for 8 h using a magnetic stirrer before hydrothermal treatment at 180°C in a Teflon-lined stainless steel autoclave for 12 h. The obtained precipitates with light yellow color were washed with purified water and dried at 100°C for 2 h.

The morphology and the average particle size were investigated using a HITACHI S-4800 scanning electron microscopy (SEM) equipped with an energy-dispersive spectrometer (EDS, Inca 400, Oxford Instruments, Abingdon, England, UK). The phase determination of the as-prepared powders was performed using an X-ray diffractometer (XRD) with Cu Kα as the X-ray source (Rigaku Miniflex-1, Shibuya-ku, Japan). Fourier-transform infrared spectroscopy (FTIR) spectra were recorded in the spectral range of 4,000 ~ 500 cm^−1^ with a spectral resolution of 4 cm^−1^ (JASCO FTIR-4100, Easton, MD, USA). Diffuse reflectance measurements (DRS) on dry powders were performed using a SCINCO S-3100 double beam spectrophotometer (Twin Lakers, WI, USA). Photoluminescence (PL) measurement was performed at room temperature using a 325-nm He-Cd laser as the excitation source.

## Results and discussion

**Figure 1:**
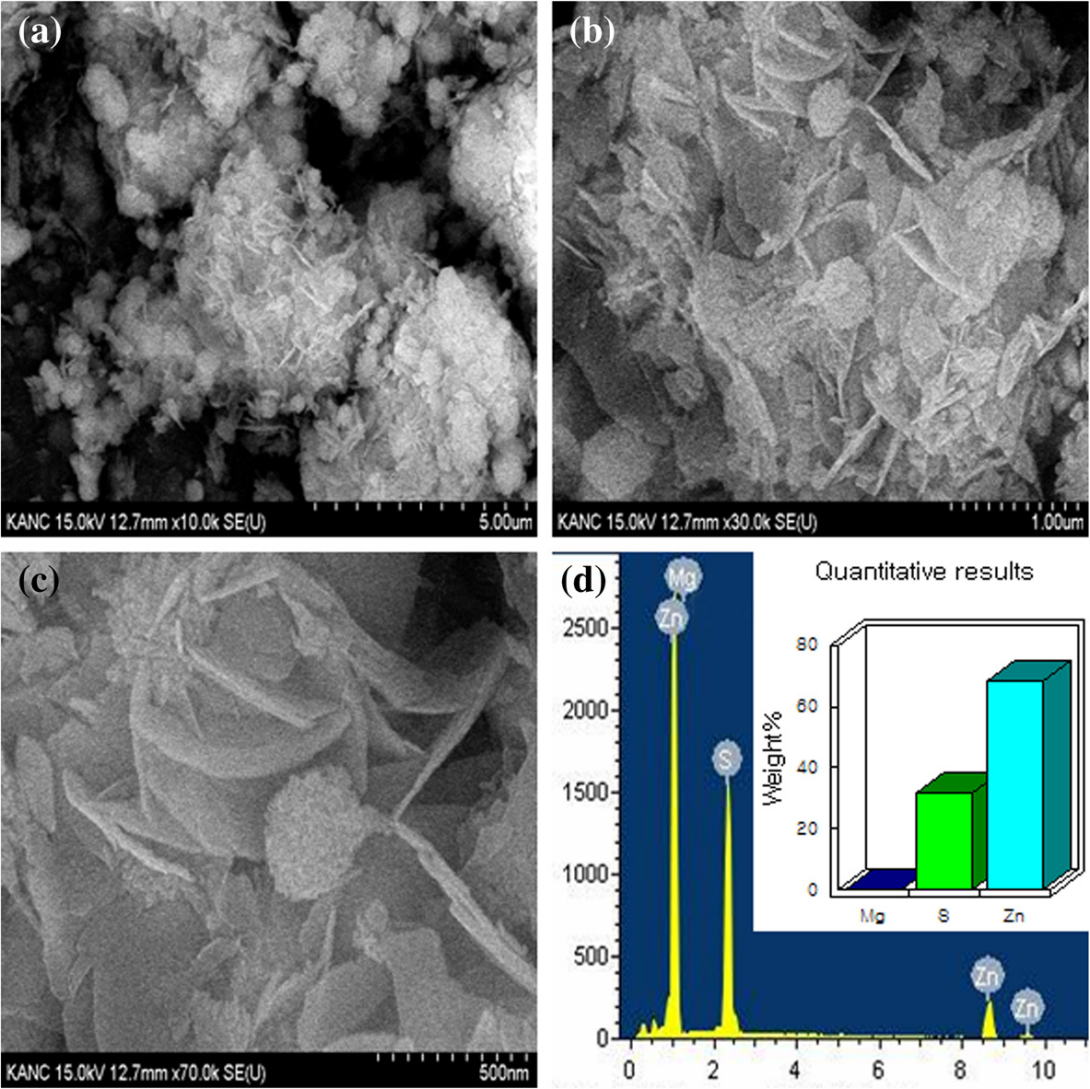
**SEM and EDS spectra of Zn**_**0.97 **_**Mg**_**0.03**_**S hierarchical nanospheres (a,b,c,d).**

Typical SEM images of Zn_0.97_ Mg_0.03_S are shown in Figure 1. Large spheres of several micrometers are clearly observed in Figure 1a. With higher magnification Figure 1b,c revealed that the individual spheres were actually assemblies of a lot of well-aligned nanosheets. The nanosheets are monolayers with a granular morphology other than smooth surface, which may imply that the nanosheets are made up of numerous well-aligned nanoparticles.

Figure 1d shows the typical EDS spectrum of Zn_0.97_ Mg_0.03_S with the characteristic peaks corresponding to the binding energy state of Zn, S, and Mg only. No other impurity peaks are detected in the spectrum, which is an indication of the chemical purity of the sample. The inset of Figure 1d gives the quantitative analysis result of the element composition in Zn_0.97_ Mg_0.03_S, which confirms that the obtained material has good stoichiometry.

**Figure 2:**
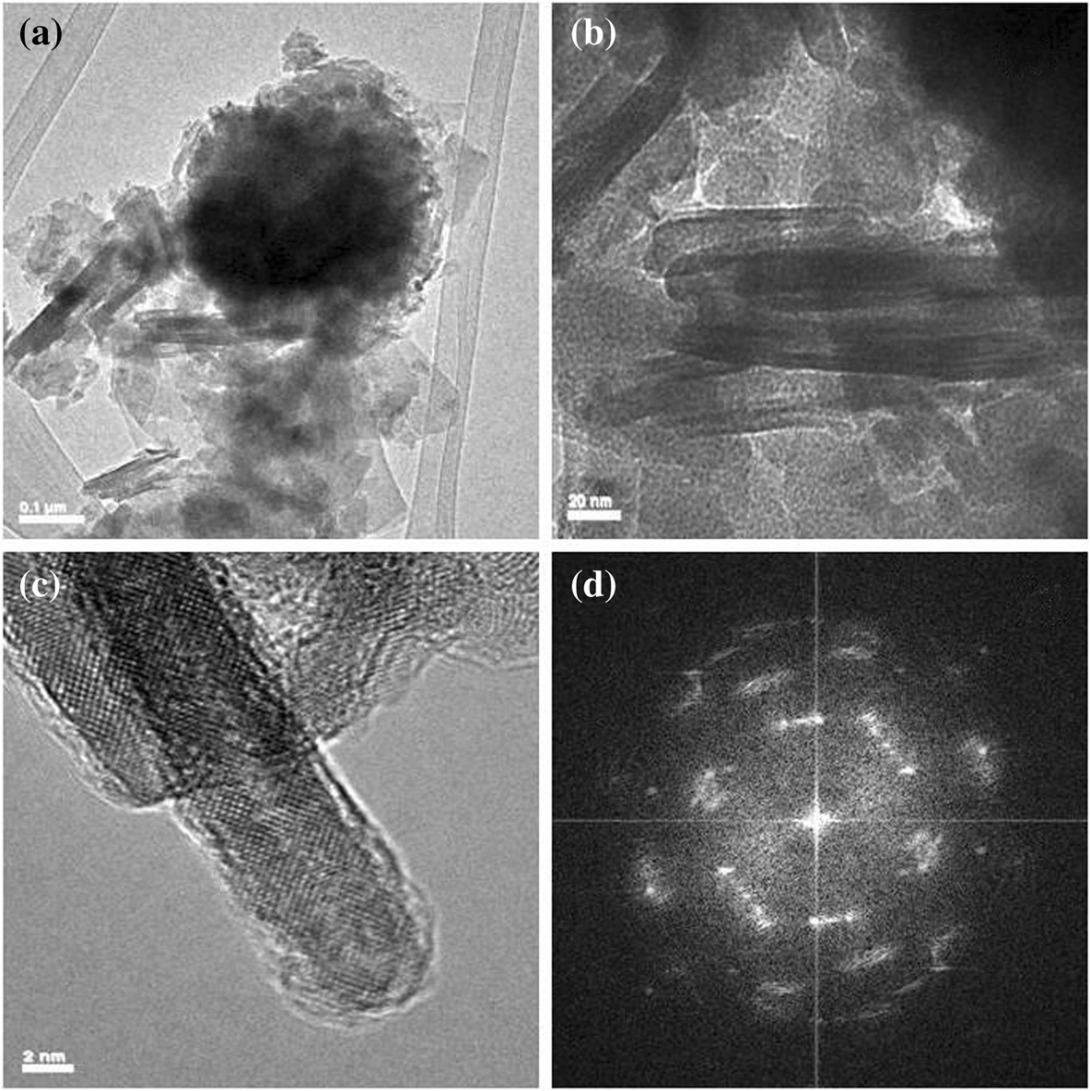
**TEM (a), HRTEMs (b) and (c), and SAED pattern (d) of Zn**_**0.97 **_**Mg**_**0.03**_**S hierarchical nanospheres.**

The microstructure of the synthesized products was further investigated by TEM and HRTEM techniques. Figure 2a is the TEM image of a randomly selected nanoparticle, which displays a sphere-like morphology. The high-resolution TEM images (Figure 2b,c) further indicate that these spheres are composed of a lot of well-aligned nanosheets. The nanosheets are 10 nm in width and 50 ~ 100 nm in length. The lattice fringes are observed to have a spacing of 0.29 nm, which are close to the interplanar spacing of the (002) plane of ZnS:Mg. The selected area electron diffraction (SAED) patterns (Figure 2d) obtained from the isolated nanosheets show the characteristic diffused electron diffraction rings of poly crystalline materials.

**Figure 3:**
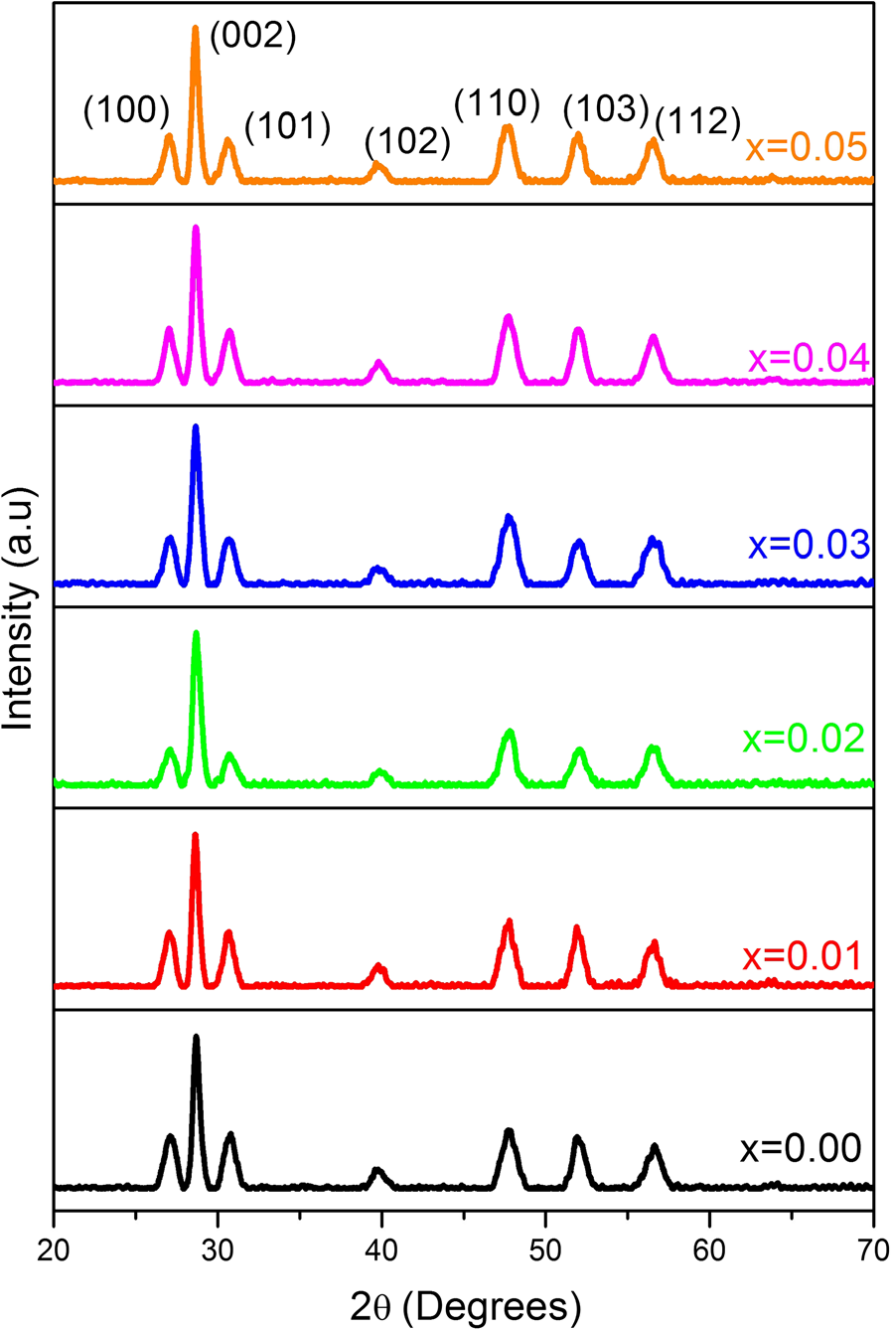
**X-ray diffraction patterns of Zn**_**1−*x***_**Mg**_***x***_**S (*x*** **= 0.0, 0.01, 0.02, 0.03, 0.04, and 0.05) hierarchical spheres.**

The X-ray diffraction patterns of Zn_1−*x*_Mg_*x*_S (*x* = 0.00, 0.01, 0.02, 0.03, 0.04, and 0.05) hierarchical spheres are shown in Figure 3. The seven broadened diffraction peaks from the left to the right corresponds to those from the (100), (002), (101), (102), (110), (103), and (11 2) lattices, respectively. The diffraction peaks of all the samples perfectly match with the wurtzite ZnS structures (standard card (ICDD 36–1450)). However, as compared to the standard diffraction spectrum, the (0 0 2) diffraction peak in Figure 3 is stronger and narrower than the other peaks, suggesting a preferential growth direction along the *c*-axis. With an increase in the doping concentration, the position of the diffraction peaks shows a slight shift to a higher diffraction angle, which can be attributed to the smaller ionic radius of Mg^2+^ (0.57 Å) as compared to Zn^2+^ (0.60 Å). The lattice parameters *a* and *c* for the wurtzite ZnS:Mg were evaluated from the (100) and (002) planes, respectively. As the Mg concentration increases, the lattice constants slightly decrease. The estimated lattice constants are *a* = 3.72 to 3.81 Å and *c* = 6.12 to 6.28 Å, and the corresponding *c/a* ratio is 1.55 to 1.62, which is slightly less than the standard value 1.638, indicating that the wurtzite Zn_1−*x*_Mg_*x*_S is under compressive strain. The average crystallite sizes of the samples were estimated using the Debye-Scherrer formula *D* = 0.89*λ*/*β*cos*θ*, where *λ* is the wavelength of the Cu Kα radiation, *β* is the FWHM of the diffraction peak, and *θ* is the diffraction angle for the (0 0 2) planes of wurtzite ZnS. The estimated crystallite sizes indicated a steady decrease of crystallite size with increasing Mg concentration in the range of 19 to 14 nm. Although no report on lattice parameter and crystallite size of the Mg-doped ZnS hexagonal nanostructures is available for comparison, similar phenomena have been reported in Mg-doped ZnO nanostructures [40].

**Figure 4:**
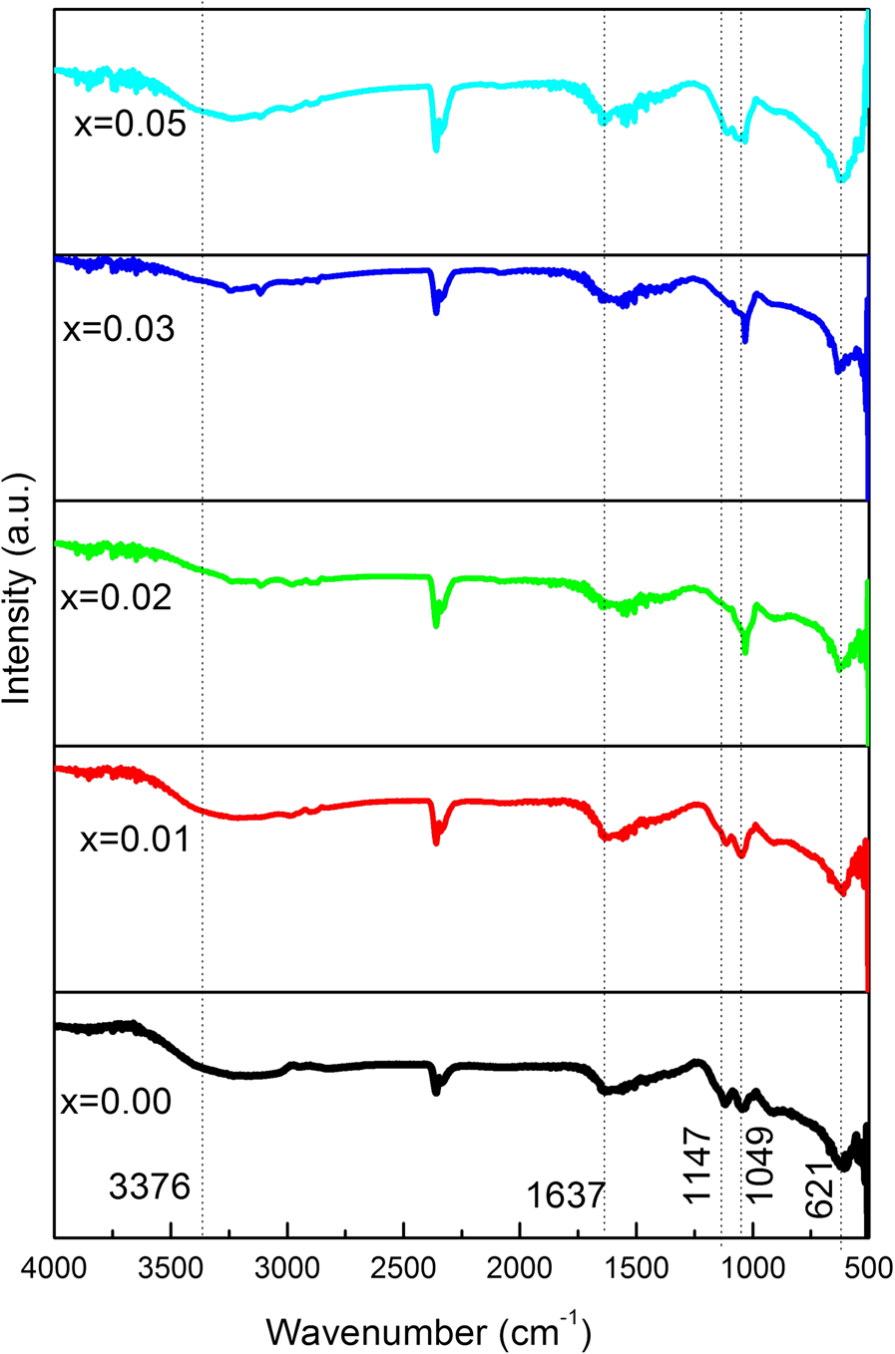
**FTIR spectra of Zn**_**1−*x***_**Mg**_***x***_**S (*x*** **= 0.00, 0.01, 0.02, 0.03, and 0.05) hierarchical spheres.**

The FTIR spectra of ZnS with different Mg doping concentrations are shown in Figure 4. The broad absorption peak around 3,376 nm is assigned to the O-H characteristic vibration resulting from small quantity of adsorbed H_2_O on the sample. The peak positions located at 1,637, 1,147, and 1,049 cm^−1^ matches with the rocking mode of NH_2_, the C = C stretching bonds, and the CH_2_ twist band, respectively [41]. The peak at 621 cm^−1^ is assigned to the Zn-S bond [22]. The close similarity of the FTIR spectra of doped and undoped samples indicates that Mg have entered the ZnS lattice substitutionally without altering the crystal structure. The above results strongly confirm that the EN molecules induced the formation of wurtzite structure through coupling with ZnS [22].

**Figure 5:**
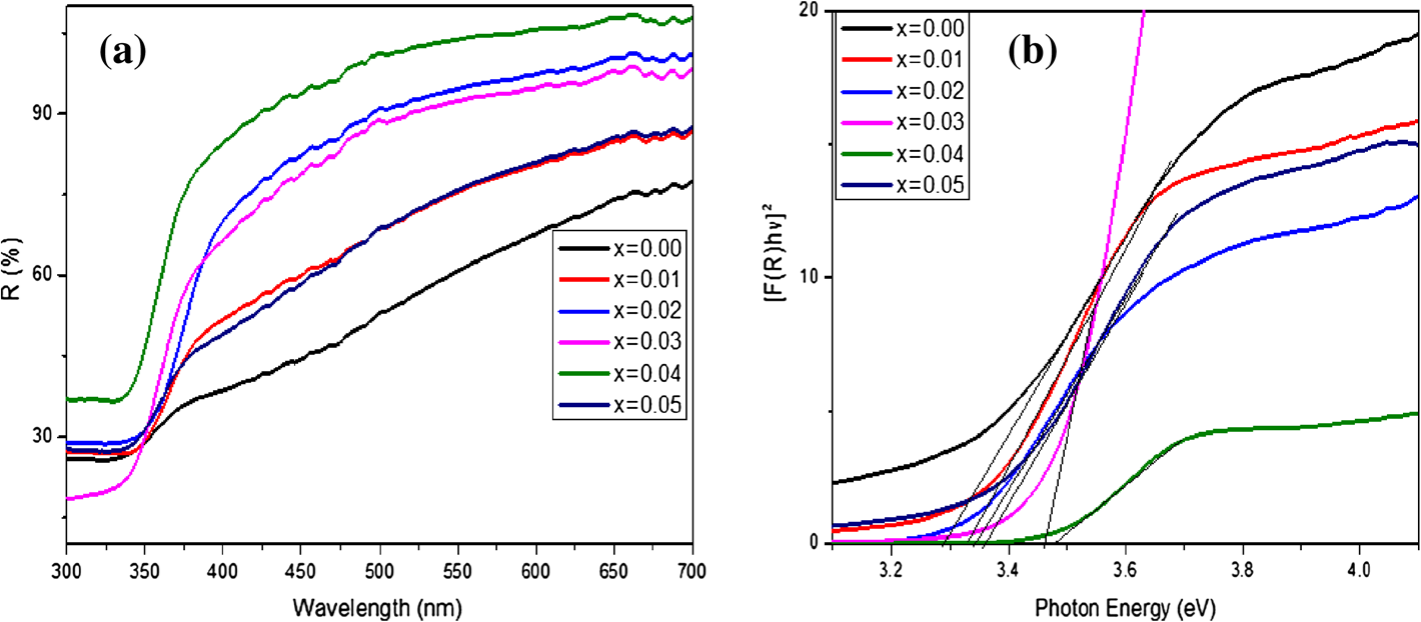
**DRS spectra (a) and Kubelka-Munk plots (b) for the band gap energy estimation for Zn**_**1−** ***x***_**Mg**_***x***_**S hierarchical spheres.**

The UV-vis DRS of Zn_1−*x*_Mg_*x*_S (*x* = 0.00, 0.01, 0.02, 0.03, 0.04, and 0.05) were taken in the range of 300 to 700 nm at room temperature as shown in Figure 5a. Careful examination of DRS reveals that the absorption edge slightly shifted towards lower wavelength as the Mg concentration increased up to 4 at %, then shifted back to higher wavelength at 5 at %. The bandgap energy of Zn_1−*x*_Mg_*x*_S was calculated by plotting a graph between the square of the Kubelka-Munk function *F*(*R*)^2^ and energy in electron volts as shown in Figure 5b [42]. From the Kubelka-Munk plots, the optical bandgap of Zn_1−*x*_Mg_*x*_S (*x* = 0.00, 0.01, 0.02, 0.03, 0.04, and 0.05) are 3.28, 3.32, 3.34, 3.46, 3.48, and 3.36 eV, respectively. The increase of bandgap for Mg-doped ZnS may be attributed to the electronegativity and ionic radius difference of Mg^2+^ and Zn^2+^ ions. Generally, the Fermi level of intrinsic ZnS is inside the conduction band, whereas that of Mg-doped ZnS could locate at a higher level due to the electrons generated by the Mg dopant. Therefore, the radiative recombination of excitons may show a larger bandgap [43]. Another observation from the bandgap study is that all samples showed smaller bandgap values than that of the bulk wurtzite ZnS, which is 3.9 eV. This red shift may be attributed to the size effect and morphology of the ZnS sample obtained under our experimental conditions. Although no report is available on wurtzite ZnS:Mg nanostructures for comparison, similar observations have been reported for hexagonal structured ZnS hierarchical microspheres [44].

**Figure 6:**
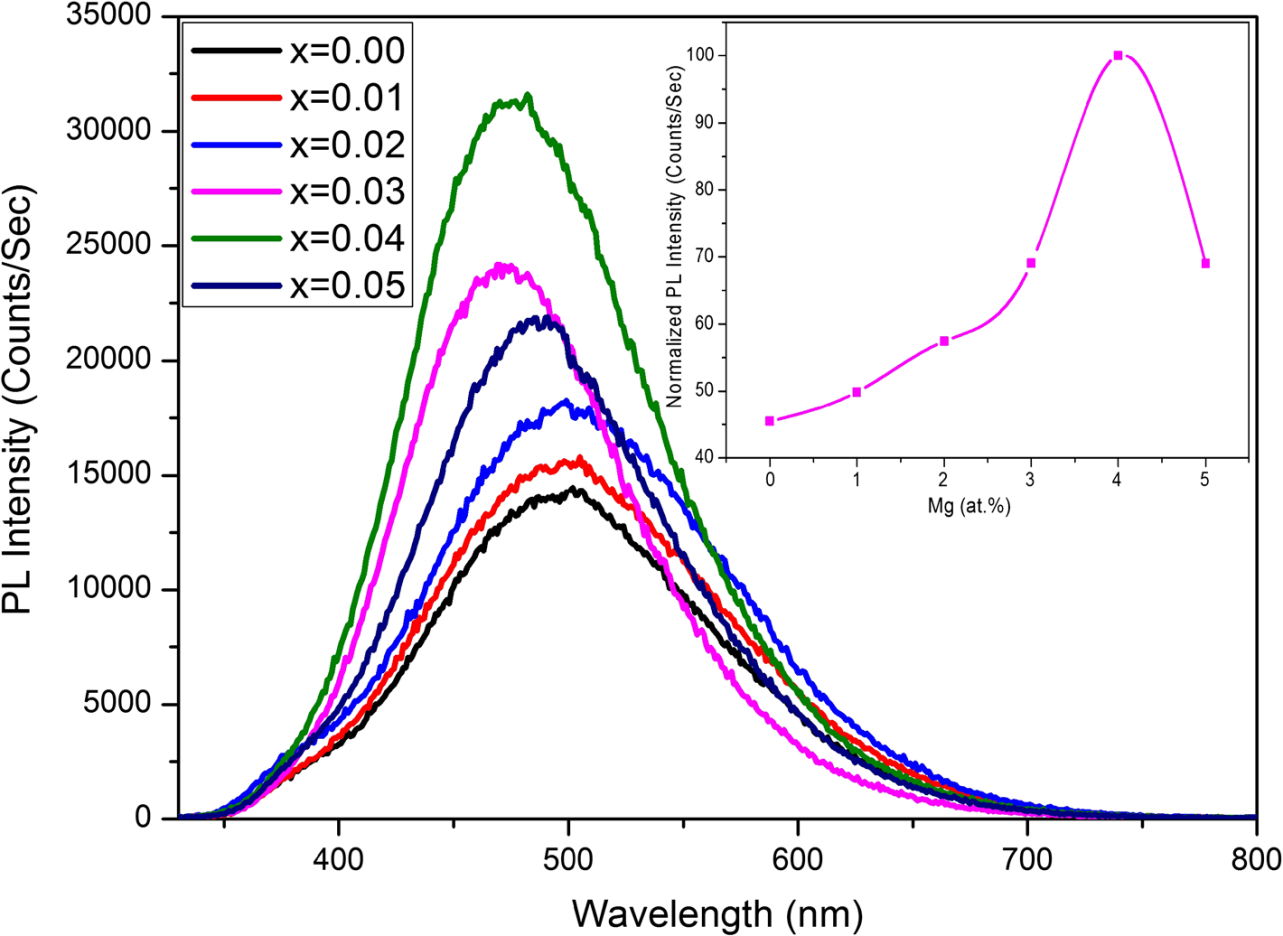
**PL spectra of Zn**_**1−*x***_**Mg**_***x***_**S (*x*** **= 0.00, 0.01, 0.02, 0.03, 0.04, and 0.05) hierarchical spheres.** The inset shows the normalized intensity as a function of Mg doping concentration.

The photoluminescence spectra of the Zn_1−*x*_Mg_*x*_S (*x* = 0.00, 0.01, 0.02, 0.03, 0.04, and 0.05) hierarchical spheres are shown in Figure 6. The emission spectra of all samples contain a broad and asymmetric emission band in the range of 350 to 700 nm. The broad emission may be due the recombination of electron-hole pairs at defect sites, which can result in a significant change of the local charge distribution and normally leads to changes in the equilibrium bond length and strong vibronic transitions [45]. It can be seen that the PL peak maximum at 503 nm of the undoped ZnS hierarchical spheres is related to the green region. In most cases, ZnS nanostructures with high crystallinity show a band edge emission about 340 to 360 nm and a broad emission band centered in the range of 420 to 450 nm that originates from the surface defect states such as sulfur vacancies [46]. The absence of blue emission, in our case, indicates the unavailability of a considerable number of sulfur vacancies to impart blue emission. Additionally, the absence of band edge emission in the present sample indicates that rather than the sulfur vacancies, some other types of defect states are presented as the origin of the green emission. Recently, a few researchers have reported green emission from undoped ZnS nanostructures. Ye et al. [47] reported PL emission peak at 535 nm in ZnS nanobelts grown by thermal evaporation technique at 1,100°C and assigned it to the elemental sulfur species. Tsuruoka et al. [48] attributed the green emission band located around 535 nm to the line or planar defects of the ZnS nanobelts fabricated using thermal evaporation technique at 800°C. Additionally, the green emission band peaked at 525 nm was suggested to be originated from the self-activated zinc vacancies of the ZnS nanostructures fabricated with solvothermal method at 160°C [49]. It was proposed that for nanoparticles with reduced size, more zinc vacancies can locate at the surface and exhibit a dominant effect as green emission in the PL spectrum. Considering the low temperature process used in our experiment and the large surface area presented on the surface of nanosheets, it is reasonable to attribute the observed green emission to zinc vacancies in ZnS nanospheres.

It is interesting to note from Figure 6 that an appreciable blue shift in the PL emission peak position (from 503 to 475 nm) is noticed with increasing Mg content. The emission peak blue shifted with Mg concentration up to 4 at %, then shifted back at higher concentration. This trend is similar with the dependence of bandgap energy on the doping concentration shown in Figure 5. Regarding the PL intensity, the inset of Figure 6 shows the normalized intensity as a function of Mg doping concentration, which also exhibits a maximum at Mg concentration of 4 at %. The blue shift and the enhancement of the PL spectrum could be caused by the generation of new radiation centers or size decrease due to Mg doping [33]. Mg ions could partially fill the tetrahedral interstitial sites or the position of Zn in the lattice of ZnS. Due to the smaller radius of Mg ions, the volume of the unit cell and the crystallite size decreased as discussed in the XRD analysis, which can lead to the blue shift of the absorption and PL spectra. When the Mg concentration is increased beyond 4 at %, the excess dopant ions could cause more deformation of the ZnS lattice that deteriorated the optical properties. Similarly, a small blue shift with Mg doping was reported in cubic structured ZnS:Mg nanoparticles [34].

**Figure 7:**
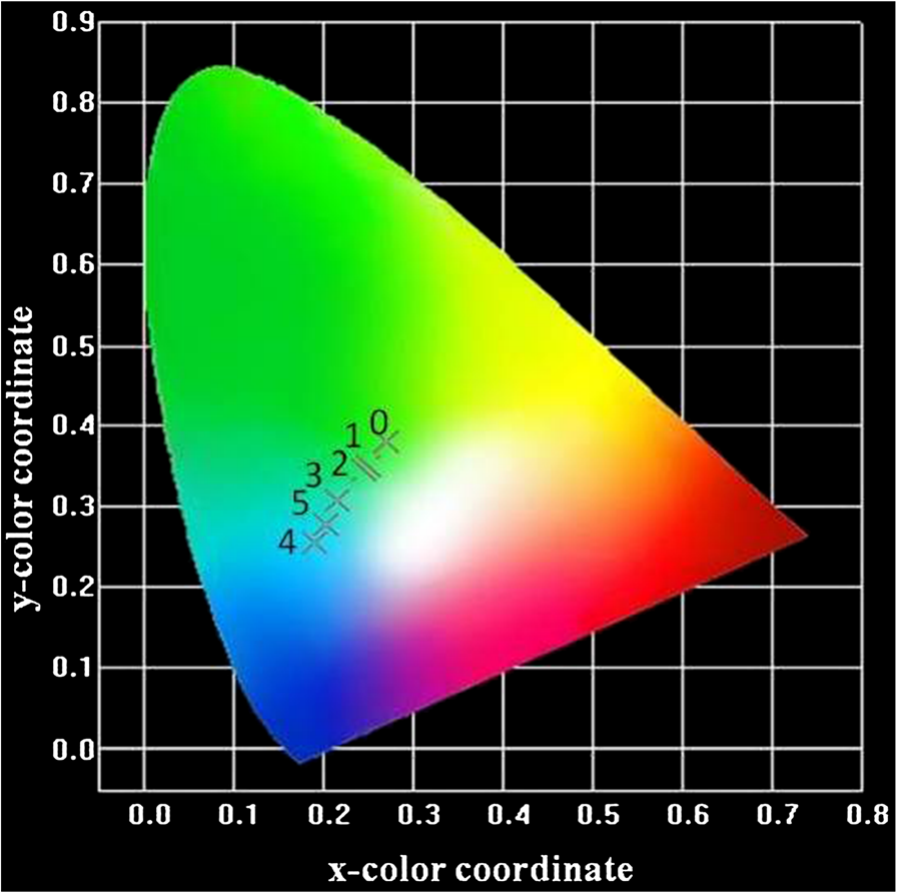
**CIE chromaticity diagram for Zn**_**1−** ***x***_**Mg**_***x***_**S hierarchical spheres.**

In order to find out the potential application of ZnS/Mg nanostructures in future white light-emitting devices (LEDs), we have calculated the CIE chromaticity coordinates for all the samples using a CIE calculation software. Figure 7 shows that the estimated CIE chromaticity coordinates are in the blue-green region next to white, which implies that by careful design and control of the composition, wurtzite Zn_1−*x*_Mg_*x*_S hierarchical spheres can be applied to the blue-green components in near UV-white LEDs.

## Conclusions

Wurtzite Zn_1−*x*_Mg_*x*_S nanosheets assembled hierarchical spheres have been synthesized using a hydrothermal approach with EN. Surface morphology studies show that the hierarchical spheres are composed of nanosheets. XRD studies showed that samples of all compositions crystallized in ZnS wurtzite structure. Widening of the bandgap was observed in Mg-doped ZnS nanostructures compared to undoped ZnS. Enhanced photoluminescence with increase in Mg doping was observed up to 4 at %. The CIE chromaticity diagram indicated that Zn_1−*x*_Mg_*x*_S with various doping concentration of Mg has potential applications for blue-green components in near UV-white LEDs.

## Competing interests

The authors declare that they have no competing interests.

## Authors’ contributions

DAR prepared the samples and took the XRD, SEM, TEM, DRS, and FTIR; DAR, DHK, and SJR collected PL data. All authors contributed to the data analysis. DAR wrote the manuscript with contributions from all authors. BWL and CL supervised the research. All authors read and approved the final manuscript.
